# 24 h severe fluid restriction increases a biomarker of renal injury in healthy males

**DOI:** 10.1007/s00421-025-05749-7

**Published:** 2025-03-07

**Authors:** Loris A. Juett, Mark P. Funnell, Harriet A. Carroll, Lewis J. James, Stephen A. Mears

**Affiliations:** 1 School of Sport, Exercise and Health Sciences, Loughborough University, Leicestershire, UK; 2 Department of Sport Science, School of Science and Technology, Nottingham Trent University, Nottingham, UK; 3 Diabetes Research Centre, NIHR Applied Research Collaboration East Midlands, University of Leicester, Leicester, UK; 4 Clinical Research Centre, Cardiovascular Research-Hypertension, Lund University, Malmö, Sweden; 5 Long Covid Scientific Consultancy, Aberdeen, UK; 6 Department for Health, University of Bath, Bath, UK

**Keywords:** Hydration, Kidney, Hypohydration, Glucose, Water

## Abstract

**Purpose:**

Exercise-induced hypohydration exacerbates biomarkers of renal injury, but studies isolating the effects of hypohydration without exercise have produced mixed findings. This study investigated the effects of 24-h severe fluid restriction on biomarkers of renal injury and glucose tolerance.

**Methods:**

Fifteen males (age: 27 ± 5 y; BMI: 24.1 ± 3.8 kg/m^2^) completed two randomised trials, involving consuming either 40 mL/kg body mass water to maintain euhydration (EU) or severe fluid restriction via limiting water consumption to 100 mL (HYP). A standardised dry food diet was consumed in both trials (~ 300 g water). At baseline and 24 h post-baseline, nude body mass, and blood and urine samples (additional urine sample at 12 h) were collected. An oral glucose tolerance test was conducted after 24-h post-baseline measurements (*n* = 12).

**Results:**

At 24 h, body mass loss (HYP: − 1.52 ± 0.34%, EU: − 0.24 ± 0.40%), plasma volume loss, serum, and urine osmolality were greater in HYP than EU (*P* ≤ 0.004). Osmolality-corrected urinary kidney injury molecule-1 (uKIM-1) concentrations were greater in HYP at 12 (HYP: 1.097 ± 0.587 ng/mOsm, EU: 0.570 ± 0.408 ng/mOsm; *P* < 0.001) and 24-h (HYP: 1.932 ± 1.173 ng/mOsm, EU: 1.599 ± 1.012 ng/mOsm; *P* = 0.01). There was no trial-by-time interactions for osmolality-corrected urinary neutrophil gelatinase-associated lipocalin concentrations (*P* = 0.781) or plasma glucose (*P* = 0.550) and insulin (*P* = 0.193) concentrations.

**Conclusion:**

Hypohydration produced by 24-h fluid restriction increased proximal tubular injury but did not affect glucose tolerance.

## Introduction

Severe fluid restriction, be it inadvertent or intentional, is commonly seen for several reasons, including lack of fluid availability, lack of thirst, to avoid urination, or to reduce body mass to ‘make weight’ in weight-category sports (Phillips et al. 1984; Smith 2006; Bottin et al. 2019). Well-controlled cross-over studies, conducted in laboratory environments, demonstrate that hypohydration produced by exercise in hot (Chapman et al. 2020; Juett et al. 2024) and temperate (Juett et al. 2021) conditions increases renal injury, compared to maintaining euhydration (Chapman et al. 2020; Juett et al. 2021). Additionally, in a case study of a mixed martial arts athlete that restricted their fluid intake (in combination with regular bouts of heat stress) for 24 h prior to a pre-competition weigh-in, acute kidney injury (AKI) was observed (Kasper et al. 2019). However, the increased renal injury documented in these studies was in the presence of combined stressors, i.e., hypohydration combined with heat stress and/or exercise. Given that exercise and/or heat induce mechanistic effects that might exacerbate the effects of hypohydration on renal injury, the isolated effect of hypohydration is of interest.

Observational evidence in humans suggests that lower water intake (Sontrop et al. 2013; Wang et al. 2021) or markers indicative of low water intake (Tasevska et al. 2016; Kuwabara et al. 2017; El Boustany et al. 2018) are associated with a higher prevalence/risk of chronic kidney disease (CKD) and/or a faster decline in renal function. Whilst causation cannot be inferred from such studies, there is causal evidence of negative effects of water restriction on renal health in animal models. García-Arroyo et al. (2020) showed that fluid restricting rats to one-third of their regular fluid intake for 22 h per day for 30 days, and only permitting rehydration with water for the remaining 2 h, increased markers of renal tubular injury, such as kidney injury molecule-1 (KIM-1), compared to controls. In humans, intervention trials that have investigated the effect of fluid restriction (in the absence of vigorous exercise and heat stress) on biomarkers of renal injury have produced mixed findings, with increases after 8 h (Noto et al. 2019), no change after 12 h (Bitker et al. 2021), and increases after 24 h (Chapman et al. 2023).

In addition to the negative effects on renal function, observational evidence suggests that low water intake is also associated with a higher type 2 diabetes risk score (Carroll et al. 2015), a higher risk of new-onset hyperglycaemia (Roussel et al. 2011), and a higher HbA1C (in males) (Carroll et al. 2016). However, these observational findings are inconsistent (Carroll et al. 2024) and could be confounded by plain water intake being positively associated with fibre intake and physical activity, and negatively associated with sugar intake. Intervention studies that have increased extracellular osmolality in healthy adults, via intravenous infusion of hypertonic saline report increased blood/plasma glucose compared to iso-osmolality (Keller et al. 2003; Jansen et al. 2019). Moreover, other intervention studies report hypohydration to impair glucose control in individuals with type 1 (Burge et al. 2001) and type 2 diabetes (Johnson et al. 2017). However, in non-clinical populations, increasing (Enhörning et al. 2019) or decreasing (Carroll et al. 2019) water intake does not appear to influence glucose control. Although, what causes the disparity in results between studies is not clear, supporting the need for further work (Carroll and James 2019).

Therefore, the main aim of the present study was to investigate the effect of 12- and 24-h severe fluid restriction on uKIM-1 and urinary neutrophil gelatinase-associated lipocalin (uNGAL) concentrations in humans. A secondary aim was to investigate the effect of this intervention on glucose tolerance. It was hypothesised that 24 h of severe fluid restriction would increase uKIM-1, but not uNGAL concentrations, compared to when euhydration was maintained with water ingestion.

## Materials and methods

### Participants

Sixteen males, with no known history of kidney issues or diabetes, participated in the present study. Participants were excluded from participation if they smoked/vaped, or if they had any current medical conditions/regularly used any medications (for example, non-steroidal anti-inflammatory drugs) that could influence the study outcomes. One subject developed an illness unrelated to the study and withdrew before completing the study, leaving 15 participants (age: 27 ± 5 y; height: 1.79 ± 0.07 m; body mass: 76.4 ± 9.3 kg; BMI: 24.1 ± 3.8 kg/m^2^). The Loughborough University Ethics Approvals (Human Participants) Sub-Committee granted this study full ethical approval (Reference: R18-P144).

### Study design

Participants visited the laboratory on five occasions: for a screening visit and two experimental trials (two visits per experimental trial). Experimental trials were performed with a randomised cross-over design and involved 24 h of either euhydration (EU; 40 mL/kg body mass water ingestion) or fluid restriction (HYP; 100 mL water ingestion) followed by an oral glucose tolerance test (OGTT). Experimental trials were separated by ≥ 7 days.

### Screening visit and pre-trial standardisation

After providing verbal and written informed consent, height and nude body mass (AFW-120 K, Adam Equipment Co., UK) were measured. If necessary, participants were familiarised to blood sampling procedures. The day prior to their first experimental trial, participants recorded their food and fluid intake, ensuring to consume ≥ 40 mL/kg body mass of non-alcoholic fluid (marked bottles were supplied to aid compliance), and avoided alcohol consumption and strenuous exercise. These were all then replicated the day before the second experimental trial. Participants were instructed to cease food and fluid consumption ≥ 10 h before arrival to all experimental trial visits. Pre-trial standardisation reminders were sent to participants two days before each experimental trial, and compliance was verbally checked when participants arrived at the laboratory.

### Experimental trials

On the morning of their first experimental trial, upon waking, participants put on a step counter wristwatch (TomTom Runner 3, TomTom, Amsterdam, The Netherlands). This would provide an approximate step count, which participants were asked to match on the first day of their second experimental trial to control habitual low-intensity physical activity between trials. Participants arrived at the laboratory between 5:45 and 9:30am (standardised within subject), provided a urine sample, and had nude body mass measured (baseline measurements). Participants then commenced seated rest and answered a series of subjective feelings questionnaires, including headache, nausea, dizziness, thirst, gastrointestinal (GI) comfort, and urge to vomit (0 = no symptom, 10 = maximum symptom). After 30 min, a blood sample was taken by venepuncture of an antecubital vein (baseline blood sample).

After eating a standardised breakfast (croissant with strawberry jam), participants were provided with urine collection equipment and their food and fluid for the rest of the day and left the laboratory. Food consisted of lunch (4 h post-baseline; cheese and butter sandwich, tortilla chips, and cereal bar), evening meal (8 h post-baseline; pizza), and evening snack (12 h post-baseline; dried apricots, raisins, and protein cereal bar). If a subject did not have access to an oven at 8 h post-baseline on their first trial, they were permitted to swap the times of their evening meal and evening snack, providing that this was matched for their second trial. The diet was designed to achieve energy balance, providing an energy intake approximately equal to estimated resting energy expenditure (Mifflin et al. 1990) multiplied by a physical activity level of 1.6 with approximately 50% of energy from carbohydrate, 35% from fat, and 15% from protein. In EU, participants consumed 7 mL/kg body mass water with each meal and 4 mL/kg body mass of water between meals, totalling 40 mL/kg body mass of water intake for the day (participants were provided with a water bottle with two markings to facilitate this, and filled bottles with tap water themselves). In HYP, participants were provided with 100 mL of water for the day, which was recommended to be consumed with their evening meal.

At home, 12 h post-baseline (just before their evening snack), participants collected and then refrigerated a urine sample. The following morning, 24 h after they first arrived at the laboratory, participants returned to the laboratory and provided a urine sample, followed by a nude body mass measurement. During seated rest, a cannula was inserted, and subjective feelings questionnaires were completed. After 30 min, a blood sample was taken from the cannula (24 h post-baseline). Participants were then instructed to ingest a solution of 75 g glucose dissolved in 250 mL water as quickly as possible, ensuring not to spill any. Once this was ingested, the cup was rinsed with 50 mL water, which was also ingested by the subject. A 10 mL blood sample was withdrawn at 15, 30, 45, 60, 90, and 120 min following the initiation of drinking. After each blood sample, the cannula was flushed with  ~ 10 mL saline.

### Sample analyses

All blood samples were  ~ 10.5 mL in volume. From this, 1 mL was dispensed into a tube containing K_2_EDTA (1.75 mg/L, Teklab, Durham, UK) and was used to determine changes in plasma volume from baseline (Dill and Costill 1974), using haemoglobin concentration (cyanmethaemoglobin method) and haemotocrit (microcentrifugation; Hawksley Microhematocrit Centrifuge, Hawksley, Worthing, UK). Another 5 mL was dispensed into a pre-chilled K_2_EDTA tube (1.6 mg/L, Sarstedt Ltd, Leicester, UK) and 4.5 mL was dispensed into a room temperature tube containing a clotting catalyst (Sarstedt Ltd, Leicester, UK). After ≥ 20 min, these two tubes were centrifuged (2200 g, 15 min, 4 °C), with the resultant plasma/serum stored at − 80 °C until analysis.

Plasma was used to determine glucose concentrations and serum was used to determine creatinine and uric acid concentrations (ABX Pentra C400; Horiba Medical, Northampton, UK), as well as osmolality by freezing point depression (Osmomat Auto, Cryoscopic Osmometer, Gonotec, Berlin, Germany). The intra-assay CV for plasma glucose, serum creatinine, and serum uric acid were 0.4, 1.6, and 0.5%, respectively. All urine samples had osmolality determined (Osmocheck; Vitech Scientific, Horsham, UK), before being aliquoted and stored at − 80 °C. They were later thawed to measure uNGAL (Human NGAL ELISA Kit, BioPorto, Hellerup, Denmark; CV: 10.0%) and uKIM-1 (KIM-1 Human ELISA Kit, Enzo Life Sciences, Lausen Switzerland; CV: 6.1%) concentrations, using commercially available ELISA kits. Plasma insulin was measured using a commercially available ELISA kit (Mercodia, Uppsala, Sweden; CV: 2.4%).

### Data analyses

Manipulating hydration status can alter the concentrations of biomarkers by concentrating/diluting blood and urine. Therefore, serum/plasma biomarkers were corrected for changes in plasma volume and urine biomarkers were corrected for urine osmolality, with results presented in both uncorrected and corrected forms. For plasma glucose and insulin concentrations, incremental (iAUC) and total (tAUC) area under the curve (Narang et al. 2020), Matsuda insulin sensitivity index (ISI; Matsuda and DeFronzo 1999), and the homeostatic model of insulin resistance (HOMA-IR2; HOMA2 calculator v2.2.3, University of Oxford) were also determined. As blood samples were not collected from two participants, and were haemolysed in one subject, all blood measures are presented as *n* = 12.

Data were analysed using IBM SPSS statistics (version 27). Shapiro–Wilk tests were used to check data for normality of distribution. Paired *t* tests or Wilcoxon signed-rank tests, depending on normality, were used to analyse data containing one factor (trial). Data containing two factors (trial × time) were analysed using a two-way repeated-measures analysis of variance. If data violated the assumption of sphericity, the Greenhouse–Geisser adjustment was used. Significant effects, which were defined as *P* ≤ 0.05, were followed up with Holm–Bonferroni corrected *t* tests or Holm–Bonferroni corrected Wilcoxon signed-rank tests, as appropriate. Parametric data are presented as (mean ± SD), and non-parametric data are presented as (median [interquartile range]).

At the time of the study, there were no published data available to inform the effect size of manipulating hydration status, in the absence of exercise, on osmolality-corrected uNGAL and uKIM-1 concentrations. Therefore, in line with studies that have found significant effects when assessing the effects of manipulating hydration status during exercise on biomarkers of renal injury (Chapman et al. 2020; Juett et al. 2021), 16 participants were recruited.

## Results

### Trial conditions

At baseline, body mass (HYP: 71.95 [69.80–79.55] kg, EU: 71.85 [69.80–79.44] kg; *P* = 0.802), serum osmolality (HYP: 289 ± 2 mOsm/ kgH_2_O, EU: 289 ± 3 mOsm/kgH_2_O; *P* > 0.999), haemoglobin (HYP: 15.5 ± 0.7 g/dL, EU: 15.6 ± 0.6 g/dL; *P* = 0.764), haematocrit (HYP: 42.8 ± 1.5%, EU: 43.1 ± 1.2%; *P* = 0.421), urine osmolality (HYP: 740 [700–825] mOsm/kgH_2_O, EU: 740 [650–875] mOsm/kgH_2_O; *P* = 0.968), and thirst (HYP: 3 ± 2, EU: 3 ± 1; *P* = 0.634) were not different between trials, suggesting that participants started both trials in a similar hydration state. The diets provided contained 11,638 ± 613 kJ energy, 336 ± 18 g carbohydrate, 112 ± 8 g fat, 98 ± 6 g protein, 20 ± 1 g fibre, 7.8 ± 0.4 g salt, and ~ 300 g water. Step counts were not different between trials [HYP: 6950 (5332–8854) steps; EU: 7327 (5538–10,221) steps; *P* = 0.975].

### Hydration status measurements

**Fig. 1:**
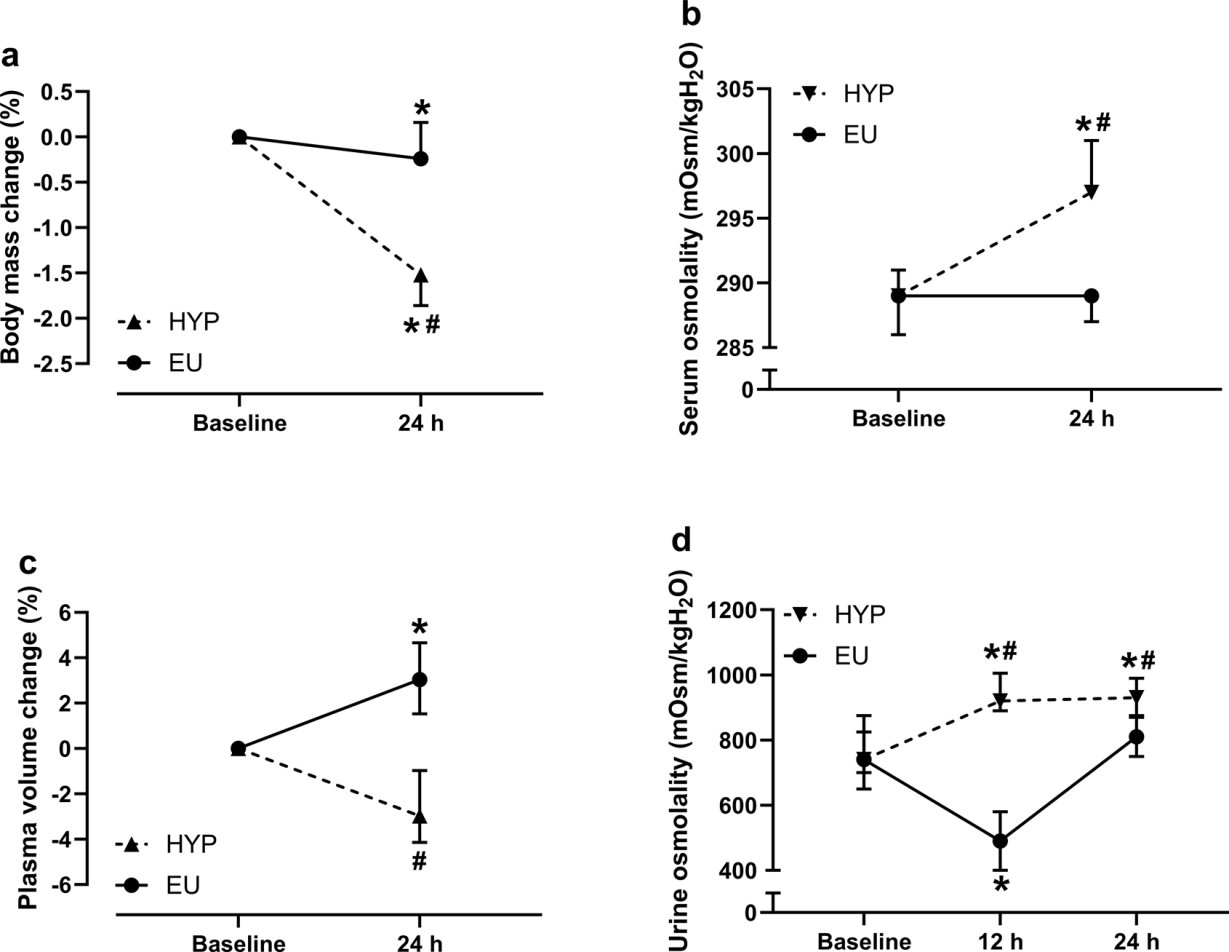
Change in body mass (**a**), serum osmolality (**b**), change in plasma volume (**c**), and urine osmolality (**d**) at baseline and 24 h post-baseline (24 h). *Indicates significantly different from baseline; # indicates significant difference between hypohydrated (HYP) and euhydrated (EU) trials. Data in (**a**) and (**b**) are presented as mean ± standard deviation, whereas data in (**c**) and (**d**) are presented as median with interquartile range

There were trial-by-time interaction effects (*P* ≤ 0.001) for changes in body mass (Fig. 1a), serum osmolality (Fig. 1b), plasma volume (Fig. 1c), and urine osmolality (Fig. 1d). Body mass decreased by a greater amount in HYP than EU (*P* < 0.001). Serum osmolality increased from baseline to 24 h in HYP (*P* < 0.001) but not EU (*P* = 0.267) and was greater in HYP than EU at 24 h (*P* < 0.001). Plasma volume increased from baseline to 24 h in EU (*P* < 0.001) but did not change in HYP (*P* = 0.136) and was greater in EU than HYP at 24 h (*P* = 0.004). Urine osmolality increased from baseline to 12 h in HYP (*P* < 0.001) and decreased in EU (*P* = 0.004), remaining elevated at 24 h in HYP (*P* = 0.001) and returning to baseline concentrations in EU (*P* = 0.135). At 12 h (*P* < 0.001) and 24 h (*P* = 0.004), urine osmolality was greater in HYP than EU.

### Biomarkers of renal injury

**Fig. 2:**
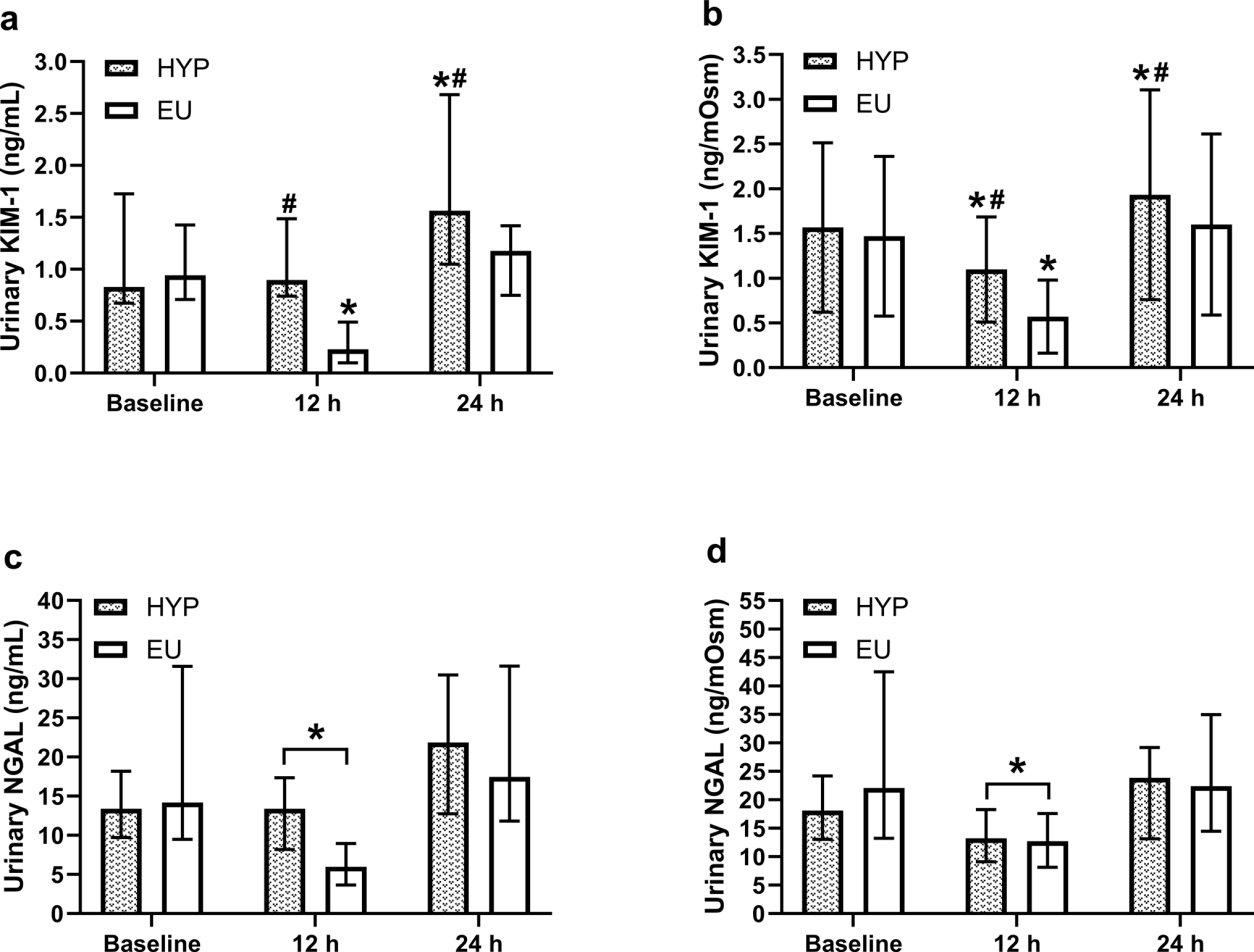
Urinary KIM-1 (**a**), osmolality-corrected urinary KIM-1 (**b**), urinary NGAL (**c**), and osmolality-corrected urinary NGAL (**d**) concentrations at baseline, 12 h post-baseline (12 h), and 24 h post-baseline (24 h). *Indicates a significant difference from baseline; #indicates a significant difference between hypohydrated (HYP) and euhydrated (EU) trials. Data in (**a**), (**c**), and (**d**) are presented as median with interquartile range, whereas data in (**b**) are presented as mean ± standard deviation

There was a trial-by-time interaction effect (*P* = 0.001) for uKIM-1 concentrations (Fig. 2a), with concentrations higher in HYP than EU at 12 h (*P* = 0.003) and 24 h (*P* = 0.004). At 12 h, uKIM-1 concentrations decreased compared to baseline in EU (*P* = 0.002) but were unchanged in HYP (*P* = 0.343). At 24 h, uKIM-1 concentrations remained elevated compared to baseline in HYP (*P* = 0.008), but not EU (*P* = 0.061). When correcting uKIM-1 concentrations for urine osmolality (Fig. 2b), the trial-by-time interaction effect remained (*P* = 0.025), with osmolality-corrected uKIM-1 concentrations 92% and 21% higher in HYP than EU at 12 h (*P* < 0.001) and 24 h (*P* = 0.01), respectively. Osmolality-corrected uKIM-1 concentrations decreased from baseline to 12 h in both trials (*P* ≤ 0.01) but were elevated at 24 h in HYP (*P* = 0.042), but not EU (*P* = 0.103) at 24 h. There was no trial-by-time interaction effect (*P* = 0.597) for uNGAL concentrations (Fig. 2c), but there was a time effect (*P* = 0.005), with uNGAL concentrations decreasing from baseline to 12 h post-baseline (*P* = 0.002) and retuning to baseline concentrations at 24 h post-baseline (*P* = 0.060). Correcting uNGAL concentrations for urine osmolality (Fig. 2d) did not alter the significance of any findings.

**Fig. 3:**
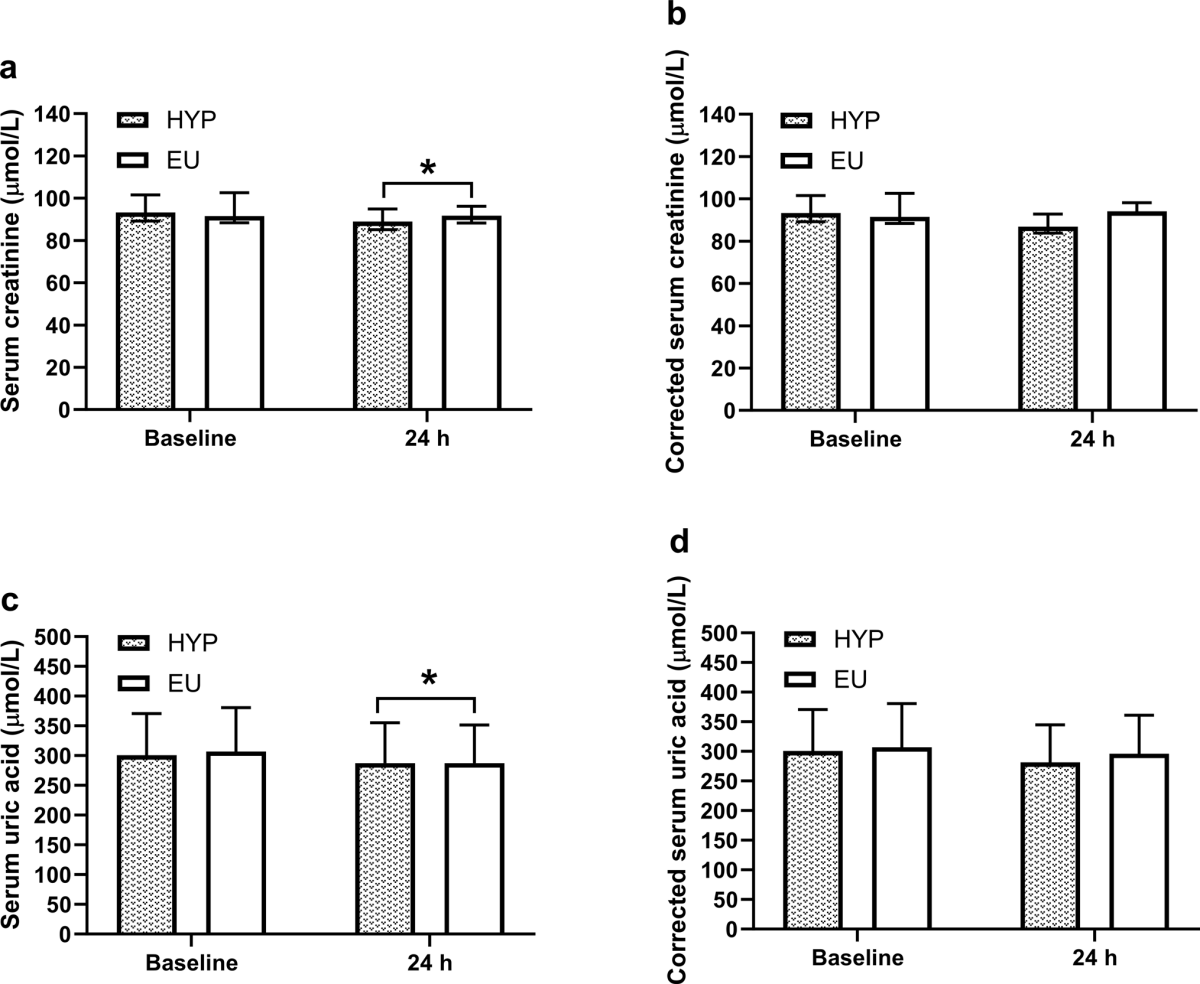
Serum creatinine (**a**), serum creatinine corrected for changes in plasma volume (**b**), serum uric acid (**c**), and serum uric acid corrected for changes in plasma volume (**d**) at baseline and 24 h post-baseline (24 h). *Indicates a significant difference from baseline. Data in (**a**) and (**b**) are presented as median with interquartile range, whereas data in (**c**) and (**d**) are presented as mean ± standard deviation

There were no trial-by-time interaction effects (*P* ≥ 0.097) for serum creatinine (Fig. 3A) or serum uric acid (Fig. 3c) concentrations, but there were time effects (*P* ≤ 0.043), with serum creatinine and serum uric acid concentrations decreasing from baseline to 24 h (*P* ≤ 0.021). Correcting serum creatinine (Fig. 3b) and serum uric acid (Fig. 3d) concentrations for changes in plasma volume removed these time effects (*P* ≥ 0.053).

### Plasma glucose and insulin concentrations

**Fig. 4:**
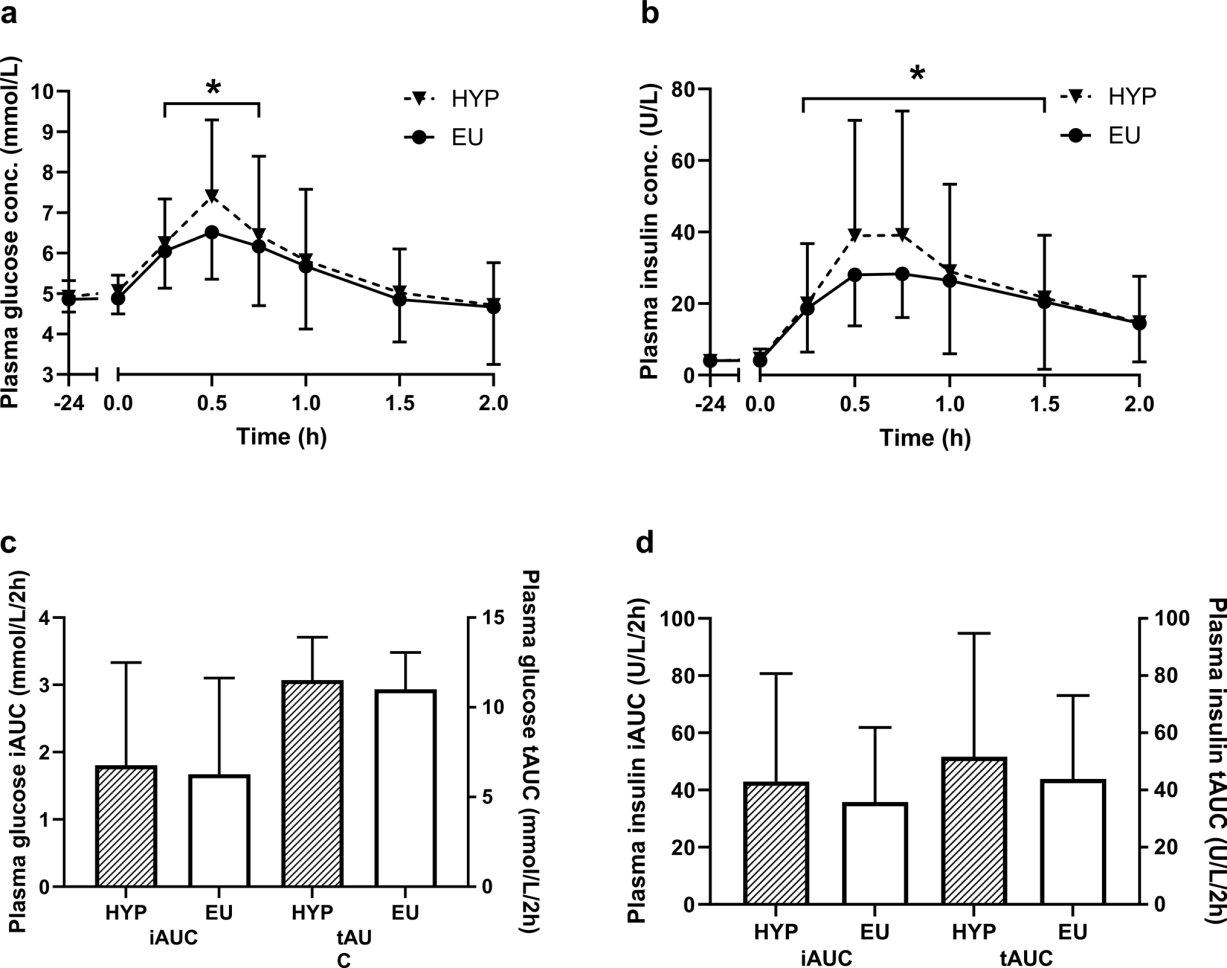
Plasma glucose concentrations (**a**), plasma insulin concentrations (**b**), total (tAUC) and incremental (iAUC) area under the curve for plasma glucose (**c**), and total (tAUC) and incremental (iAUC) area under the curve for plasma insulin (**d**). *Indicates a significant difference from 24 h post-baseline (0.0 h). Data are presented as mean ± standard deviation

There was no trial-by-time interaction effect (*P* = 0.550) for plasma glucose concentrations (Fig. 4a). There was a time effect (*P* < 0.001), with plasma glucose concentrations increased 15 (*P* < 0.001), 30 (*P* < 0.001), and 45 (*P* = 0.01) min into the oral glucose tolerance test, compared to baseline. Correcting plasma glucose concentrations for changes in plasma volume did not alter the significance of any findings. There was no trial-by-time interaction effect for plasma insulin concentrations (*P* = 0.193; Fig. 4b). There was a time effect, with plasma insulin concentrations increased above baseline from 15 to 90 min (*P* < 0.001). Correcting plasma insulin concentrations for changes in plasma volume did not alter the significance of any findings. There was no difference between trials for the tAUC for plasma glucose concentrations (*P* = 0.286; Fig. 4c), plasma glucose concentrations corrected for changes in plasma volume (*P* = 0.895), plasma insulin concentrations (*P* = 0.117; Fig. 4d), or plasma insulin concentrations corrected for changes in plasma volume (*P* = 0.182). There was no difference between trials for the iAUC for plasma glucose concentrations (*P* = 0.659; Fig. 4c), plasma glucose concentrations corrected for changes in plasma volume (*P* = 0.628), plasma insulin concentrations (*P* = 0.099; Fig. 4d), or plasma insulin concentrations corrected for changes in plasma volume (*P* = 0.099). There was no difference between trials for Matsuda ISI (HYP: 12.7 ± 5.4, EU: 14.0 ± 5.4; *P* = 0.208) or HOMA-IR2 [HYP: 0.41 (0.39–0.57), EU: 0.44 (0.40–0.55); *P* = 0.611].

### Perceptual measures

There was a trial-by-time interaction effect (*P* = 0.001) for thirst (Table 1), with thirst greater in HYP than EU at 24 h (*P* < 0.001). There were no trial-by-time interaction effects (*P* ≥ 0.060) for headache, nausea, dizziness, GI comfort, or urge to vomit (Table 1).

**Table 1:** Subjective feelings questionnaires scores at baseline and 24 h post-baseline (24 h)

	HYP		EU	
	Baseline	24 h	Baseline	24 h
Thirst (0–10)	3 ± 2	6 ± 2 **#**	3 ± 1	4 ± 1
Headache (0–10)	0 [0–1]	2 [1–3]	0 [0–0]	0 [0–1]
Nausea (0–10)	0 [0–0]	0 [0–1]	0 [0–0]	0 [0–0]
Dizziness (0–10)	0 [0–0]	0 [0–2]	0 [0–0]	0 [0–0]
Gi comfort (0–10)	0 [0–1]	0 [0–2]	0 [0–2]	0 [0–1]
Urge to vomit (0–10)	0 [0–0]	0 [0–0]	0 [0–0]	0 [0–0]

**#**Indicates a significant difference between hypohydrated (HYP) and euhydrated (EU) trials. Data are presented as either mean ± standard deviation or median [interquartile range]

## Discussion

The primary aim of the present study was to investigate the effect of severe fluid restriction on uKIM-1 and uNGAL concentrations in humans. In agreement with our hypothesis, uKIM-1 concentrations were greater after 12- and 24-h fluid restriction compared to euhydration. These effects remained significant when uKIM-1 concentrations were corrected for urine osmolality, suggesting that the increased uKIM-1 concentrations were not simply due to urine concentration, but were due to increased production of KIM-1. However, fluid restriction did not influence uNGAL concentrations. Therefore, this study presents data demonstrating that severe fluid restriction, in the absence of strenuous exercise, appears to increase proximal tubular injury.

uKIM-1 expression is increased in response to proximal tubular injury (Ichimura et al. 1998; Han et al. 2002; Kashani et al. 2017), whereas an increase in uNGAL is thought to be mainly due to injury to the distal nephron (Paragas et al. 2011; Helanova et al. 2014; Bongers et al. 2017, 2018). Therefore, the greater concentrations of osmolality-corrected uKIM-1 at 12 and 24 h post-baseline when participants were fluid restricted, and lack of differences between trials for osmolality-corrected uNGAL concentrations, suggest that the site of increased renal injury was the proximal tubules. This finding is consistent with the recent studies that showed hypohydration induced by fluid restriction during exercise increased osmolality-corrected uKIM-1 (Juett et al. 2021) and osmolality-corrected urinary insulin-like growth factor-binding protein 7 (Chapman et al. 2020) concentrations, indicating an increase in proximal tubular injury. The findings from the present study are also in line with recent work by (Chapman et al. 2023), who reported an increase in osmolality-corrected uKIM-1 concentrations but no change in osmolality-corrected uNGAL concentrations in males after 24-h fluid restriction, indicating that fluid restriction increased proximal tubular injury.

The fluid restriction in the present study caused a reduction in plasma volume (albeit small and not statistically significant) and an increase in serum osmolality, indicating intracellular dehydration (Cheuvront and Kenefick 2014; James et al. 2019). This is where an increase in serum osmolality causes movement of water out of the intracellular compartment by osmosis, leading to cell shrinkage and the subsequent release of the hormone arginine vasopressin (AVP). An increase in circulating AVP is thought to be a key mechanism by which hypohydration may increase renal injury (Sugiura et al. 1999; Roncal-Jimenez et al. 2015; García-Arroyo et al. 2017; Mansour et al. 2019; Butler-Dawson et al. 2020). Though it is a limitation of the present study that AVP (or it is more stable surrogate: copeptin) was not measured, an increase in serum osmolality is the primary stimulus for the release of AVP, and thus, AVP concentrations have been shown to correlate with serum and urine osmolality (Robertson et al. 1973). Additionally, previous work using a similar fluid restriction protocol observed robust increases in copeptin within 24 h (Carroll et al. 2019). Therefore, given that serum osmolality was greatly increased with fluid restriction, it is likely that an increase in circulating AVP played a role in the increased renal injury documented in the present study.

The increase in serum osmolality may also contribute to renal injury via increased activity of the polyol–fructokinase pathway (Ruepp et al. 1996), which is an energetically costly pathway that can result in ATP depletion and subsequent renal injury (Cirillo et al. 2009; Roncal-Jimenez et al. 2014). In the kidneys, fructokinase is thought to be confined to the proximal tubules (Burch et al. 1980; Diggle et al. 2009), which is interesting given that fluid restriction appeared to increase proximal tubular injury in the present study. However, this is speculative as the evidence for the role of polyol–fructokinase pathway in renal injury derives from animal models and in vitro experiments on human cells, with no known biomarker capable of quantifying the activation of this pathway in vivo in humans (Chapman et al. 2020).

A third potential mechanism via which hypohydration may increase renal injury is by hyperuricemia (Roncal-Jimenez et al. 2015, 2018). However, in the present study, there was no difference between trials with regards to serum uric acid concentrations. Therefore, this mechanism is likely to be more relevant to exercise-induced hypohydration, where muscle damage and a significant decrease in renal blood flow (Poortmans 1984) may increase uric acid production and impair uric acid excretion, respectively (Knochel et al. 1974; Roncal-Jimenez et al. 2015).

Whilst osmolality-corrected uKIM-1 concentrations were greater after 12 h and 24  h of fluid restriction, compared to when euhydration was maintained, it was surprising that the difference between trials was greater and more significant after 12 h than after 24 h. This pattern was the same with regards to urine osmolality, as reported in another previous study (James and Shirreffs 2013), suggesting that the process of urine concentration may have increased renal injury (Bouby et al. 1990; Sugiura et al. 1999; Clark et al. 2016; Wagner et al. 2022). It seems likely that the greater difference between trials in urine osmolality at 12 h than 24 h may have been due to a greater difference in AVP concentrations at 12 h (Robertson et al. 1973). Water ingestion in the euhydrated trials may have acutely suppressed AVP concentrations via oropharyngeal neural and/or gastric neuroendocrine mechanisms (Geelen et al. 1984), an effect that would not have been present at 24 h (after an overnight fast from food and water).

The lack of blood sampling and hormone analysis at 12 h makes it difficult to explain the decrease in osmolality-corrected uKIM-1 concentrations from baseline to 12 h with fluid restriction (albeit less than when euhydration was maintained) and that osmolality-corrected uNGAL concentrations decreased from baseline to 12 h. These results suggest that biomarkers of renal injury may show a circadian pattern, with less renal injury in the evening than the morning. Perhaps, circadian fluctuations in hormones and/or being fed versus fasted play a role in the differences between renal injury markers over the day. Indeed, it has previously been shown that plasma AVP concentrations are greater in the night and early morning than in the day and evening (George et al. 1975). However, circadian fluctuations in renal injury, and the potential mechanisms responsible, require further research.

The greater concentrations of osmolality-corrected uKIM-1 after 12 and 24 h of severe fluid restriction could have implications for individuals with an extremely low fluid intake, but the long-term consequences are not well understood and require further investigation. Two human randomised-controlled trials have shown no long-term effects of increasing water intake on kidney function (Spigt et al. 2006; Clark et al. 2018). However, the difference in water intake between the intervention and control trials was less than desired in these studies (Spigt et al. 2006; Clark et al. 2018). Given this, and the findings from the present study, it could be speculated that increasing the fluid intake of individuals with low water/fluid intake could have benefits for renal health. Whilst these two long-term RCTs did exclude individuals with a self-reported fluid intake of > 2 L (Spigt et al. 2006)/ ≥ 10 cups per day (Clark et al. 2018) or a 24-h urine volume of ≥ 3 L (Clark et al. 2018), these criteria could be stricter for future studies to target individuals that severely restrict their fluid intake. Indeed, observational evidence suggests that the potential benefits of increasing water intake on renal function may start to diminish the more water intake increases (Tasevska et al. 2016; Kuwabara et al. 2017; Wang et al. 2021). Thus, it seems that individuals with very low water/fluid intake likely stand to benefit the most from an increase in water intake. Additionally, another scenario that requires further investigation is the long-term consequences of athletes restricting fluid intake (often in combination with exercise and heat stress) to elicit a drastic reduction in body mass (Hillier et al. 2019; Kasper et al. 2019) particularly given the anecdotal reports of mixed martial arts athletes suffering with kidney issues (Kasper et al. 2019).

A secondary aim of the present study was to investigate the effect of fluid restriction on glucose tolerance. In agreement with the findings of Carroll et al. (2019), 24 h of fluid restriction did not affect glucose tolerance. However, the findings from these studies are in contrast with work by Jansen et al. (2019), who demonstrated that inducing hyperosmolality (by infusing hypertonic saline) induced a greater glycaemic response to an OGTT than when iso-osmolality was maintained (by infusing isotonic saline). There are several potential explanations for the contrasting findings between these studies. The hyperosmolality induced in Jansen et al. (2019) was greater than in the present study and Carroll et al. (2019). Furthermore, this hyperosmolality in Jansen et al. (2019) occurred alongside an increase in plasma volume, due to the use of hypertonic saline. Whilst this allowed for the effects of hyperosmolality and an increase in circulating copeptin to be isolated from the renin–angiotensin–aldosterone system (RAAS), hyperosmolality would typically be accompanied by hypovolaemia (Cheuvront and Kenefick 2014). If this severe hyperosmolality had been achieved by fluid restriction, the likely large accompanying decrease in plasma volume may have increased RAAS activation (Cheuvront and Kenefick 2014). Therefore, it is uncertain how applicable the results from Jansen et al. (2019) are to more typical causes of hypohydration (like reduced fluid intake).

Nonetheless, if increased AVP impairs glucose regulation, then the present study and that of Carroll et al. (2019) should have observed this. Carroll et al. (2019) observed a 14 pmol/L increase in copeptin concentrations in the fluid restriction trial, and given the similarity in study design (i.e., 24-h fluid restriction), a comparable increase would be expected in the present study. These studies combined suggest that mild hypohydration (1–2% body mass) does not affect glucose regulation in healthy individuals. Although, perhaps, the time of day of testing might have influenced the responses, as the present study and that of Carroll et al. (2019) performed the OGTT in the morning after an overnight fast, which may have attenuated differences in circulating copeptin between trials. Evidence for this theory comes from Enhörning et al. (2019), who showed that copeptin decreased more, 90  min after an acute water load than after one week of fluid loading followed by an overnight fast. Given the greater differences in urine osmolality between trials at 12  h, compared to 24 h in the present study, and therefore potentially greater differences in copeptin, it would have been interesting to have performed the OGTT in the evening. As glucose excursions are more pronounced later in the day (Poggiogalle et al. 2018), this is something that should be the focus of future studies. Finally, as investigating glucose tolerance was a secondary aim of the present study, it was not powered to detect differences in plasma glucose [although Carroll et al. (2019) was powered for *n* = 16]. Jansen et al. (2019) had a much larger sample size (60 participants) than the present study and Carroll et al. (2019). Nonetheless, the results of the present study and those of Carroll et al. (2019) suggest that low levels of hypohydration caused by reduced fluid intake do not substantially affect glycaemic control.

It is a limitation of the present study that females were not included, particularly as the relationship between hydration status, sex hormones, and kidney injury biomarkers is relatively unknown. To highlight the need for inclusion, a previous fluid restriction study found similar differences in kidney biomarker responses in both males and females but when these biomarkers were corrected for urine osmolality and creatinine only differences in males remained (this also raises an important consideration when processing biomarker data for males and females, (Chapman et al. 2023). Although not measured, it was suggested that this may be due to the differing levels of circulating concentrations of AVP/copeptin [lower in females for maintenance of the same serum osmolality (Giersch et al. 2020)]) and a reduced sensitivity of AVP release to hypohydration in females (Stachenfeld et al. 2001). Given the important role of AVP in the development of kidney injury, future studies should involve sex as a biological variable (as the results are not similar between sexes) and should try, where possible, to measure AVP/copeptin to fully explore this potential mechanism. In terms of glycaemic control and hydration status, our null results are concordant with Carroll et al. (2019) who included both men (*n* = 8) and women (*n* = 8) and used a similar design. However, in combination with sex differences in circulating AVP and in light of previous comments related to the timing of OGTTs, AVP fluctuations throughout the day and influence of the menstrual cycle, further investigation may be required to fully explore the impact of sex.

In conclusion, the results from the present study suggest that severe fluid restriction in the absence of exercise and/or heat exposure increased proximal tubular injury in males, compared to when maintaining euhydration. This increase in proximal tubular injury was likely mediated by hyperosmolality and subsequent AVP release. However, the long-term effects of an acute increase in a biomarker of proximal tubular injury are not well understood and require further investigation.

## Data Availability

Data for the study is available upon reasonable request.

## Abbreviations

AKI: Acute kidney injury
ANOVA: Analysis of variance
ATP: Adenosine triphosphate
AVP: Arginine vasopressin
BMI: Body mass index
CKD: Chronic kidney disease
CV: Coefficient of variation
GI: Gastrointestinal
HOMA-IR: Homeostatic model of insulin resistance
iAUC: Incremental area under the curve
KIM-1: Kidney injury molecule-1
NGAL: Neutrophil gelatinase-associated lipocalin
OGTT: Oral glucose tolerance test
RAAS: Renin–angiotensin–aldosterone system
RCT: Randomised-controlled trials
SD: Standard deviation
tAUC: Total area under the curve
